# Real-world practice of idiopathic pulmonary fibrosis: Results from a 2000–2016 cohort

**DOI:** 10.1515/med-2023-0852

**Published:** 2023-11-11

**Authors:** Ying-Tso Wang, Te-Chun Shen, Cheng-Li Lin, Chih-Yen Tu, Te-Chun Hsia, Wu-Huei Hsu

**Affiliations:** Department of Laboratory Medicine, China Medical University Hospital, Taichung, Taiwan; Division of Pulmonary and Critical Care Medicine, Department of Internal Medicine, China Medical University Hospital, No. 2 Yu-De Road, Taichung 404, Taiwan; School of Medicine, China Medical University, Taichung, Taiwan; Division of Critical Care Medicine, Chu Shang Show Chwan Hospital, Nantou, Taiwan; Management Office for Health Data, China Medical University Hospital, Taichung, Taiwan

**Keywords:** idiopathic pulmonary fibrosis, comorbidity, progression, prognosis

## Abstract

The study aimed to investigate comorbidities, major adverse respiratory events, and mortality in patients with idiopathic pulmonary fibrosis (IPF). We established an IPF cohort and a comparative cohort matched for sex, age, and the date of IPF diagnosis. We recorded the most frequent comorbidities, the proportions, and time durations to the episode of major adverse respiratory events and death. Both cohorts were followed up to the end of 2016. We included 921 patients in the IPF cohort and 3,677 individuals in the comparative cohort. Comorbidities associated with IPF included pulmonary hypertension, chronic obstructive pulmonary disease, heart failure, asthma, and gastroesophageal reflux disease. The IPF cohort was more likely to have pneumonia (47.6 vs 12.0%), acute respiratory failure (17.8 vs 4.30%), chronic respiratory failure (4.23 vs 0.63%), and death (36.3 vs 15.0%) than the comparative cohort. The time durations to the first episode of pneumonia, acute respiratory failure, chronic respiratory failure, and death were 2.09 ± 2.98, 3.12 ± 3.62, 3.20 ± 4.03, and 3.27 ± 3.03 years in the IPF cohort. In conclusion, patients with IPF had significant comorbidities, particularly pulmonary and cardiovascular comorbidities. The duration from diagnosis to the major adverse respiratory events or death was short.

## 1 Introduction

Idiopathic pulmonary fibrosis (IPF) has emerged as an important public health problem. The incidence has been reported to be 5–15 per 100,000 per year and appears to be increasing [1]. IPF is a fibrosing interstitial pneumonia of unknown cause that is associated with typical features of usual interstitial pneumonia. It occurs primarily in older people and is presented by progressive worsening of dyspnea and lung function. Most importantly, IPF has a very poor prognosis with a median survival of 3–5 years [2] owing to its unknown pathophysiology, progressive nature, lack of curative treatment, delayed diagnosis, effect of chronic hypoxemia, and potential for severe complications and comorbidities.

Although IPF is a disease of unknown etiology, previous studies have identified several associated factors. Genetics, smoking, environmental exposure, microbes, and comorbidities may be associated with the development of IPF. Respiratory and cardiovascular comorbidities may play a major role in the development of IPF [3–5]. However, the precise pathophysiology is largely inconsistent. In addition, the outcomes of IPF are unpredictable, ranging from rapid progression and death within a few months to prolonged stability. The present study aimed to investigate the most frequent comorbidities and the proportions and time durations to pneumonia, acute respiratory failure, chronic respiratory failure, and mortality in patients with IPF.

## 2 Materials and methods

### 2.1 Data source

We utilized the Longitudinal Health Insurance Database, a subset of the National Health Insurance Research Database in Taiwan, which recruited 2,000,000 persons and contained their detailed medical information between 1996 and 2016. The database was managed and updated by the Ministry of Health and Welfare of Taiwan.


**Institutional review board statement:** The study was conducted in accordance with the Declaration of Helsinki, and approved by the Ethics Committee of China Medical University Hospital (CMUH-107-REC2-181).
**Informed consent statement:** Informed consent was waived due to the data extracted from the National Health Insurance Database, which provides only comprehensive de-identified healthcare information.

### 2.2 Study cohorts

We established an IPF cohort that included adult patients newly diagnosed with IPF (International Classification of Diseases [ICD] codes 516.3 and J84.112) between 2000 and 2016. Similar methods have been reported elsewhere [6,7]. The baseline comorbidities were traced and identified from 1996 to the date of the IPF diagnosis (index date), including several respiratory and non-respiratory comorbidities. In addition, we established a comparative cohort that included adults without IPF at 1:4 ratio and matched for sex, age, and the index date. Both cohorts were followed up until withdrawal from the insurance system, death, or the end of 2016, whichever came first. We also identified the occurrence of major adverse respiratory events, including pneumonia, acute respiratory failure, and chronic respiratory failure.

### 2.3 Statistical analysis

Chi-squared test was used to examine the proportion distribution of age group, gender, and comorbidity; student’s *t*-test was applied to compare mean age in the two cohorts. The estimation of cumulative incidence of death in IPF and comparison cohorts was performed by the Kaplan–Meier method. A log-rank test was utilized to determine the significance. Univariable and multivariable logistic regression analyses were adopted to calculate the crude and adjusted odds ratios (ORs) and 95% confidence intervals (CIs). Furthermore, the Chi-squared test was used to compare the proportions of major adverse respiratory events and death and the Student’s *t*-test to compare time durations to the first episode of major adverse respiratory events and death between the IPF cohort and the control during the follow-up period. Data analysis for this study was conducted using the SAS statistical software (Version 9.4 for Windows; SAS Institute, Inc., Cary, NC, USA). Statistical significance in this study was set as a *p*-value <0.05.

## 3 Results

We included 921 patients in the IPF cohort and 3,677 individuals in the comparative cohort during the study period (Table 1). The mean age was 69.7 (standard deviation [SD] = 14.2) years in the IPF cohort and 69.5 (SD = 14.1) years in the comparative cohort. The male gender was predominant (58.4%) in the IPF and comparative cohorts. The IPF cohort significantly demonstrated respiratory comorbidities, including chronic obstructive pulmonary disease (COPD, 66.9 vs 21.6%, *p* < 0.001), asthma (34.7 vs 12.4%, *p* < 0.001), pulmonary hypertension (4.02 vs 0.19%, *p* < 0.001), and pulmonary embolism (0.76 vs 0.22%, *p* = 0.01), and non-respiratory comorbidities, including ischemic heart disease (38.9 vs 29.9%, *p* < 0.001), gastroesophageal reflux disease (GERD, 24.2 vs 13.1%, *p* < 0.001), chronic liver disease (23.1 vs 20.0%, *p* = 0.04), heart failure (22.8 vs 8.46%, *p* < 0.001), and atrial fibrillation (6.73 vs 4.22%, *p* = 0.001), than the comparative cohort.

**Table 1:** Baseline characteristics of individuals with and without IPF

	IPF				
	No		Yes		
	*N* = 3,677		*N* = 921		
	*n*	%	*n*	%	*p*-value^†^
**Age**					0.96
20–49	391	10.6	95	10.3	
50–64	969	26.4	243	26.4	
≥65	2,317	63.0	583	63.3	
Mean ± SD	69.5	±14.1	69.7	±14.2	0.66
**Sex**					0.97
Women	1,532	41.7	383	41.6	
Men	2,145	58.3	538	58.4	
**Comorbidity**					
**Respiratory**					
Asthma	457	12.4	320	34.7	<0.001
COPD	794	21.6	616	66.9	<0.001
Pulmonary hypertension	7	0.19	37	4.02	<0.001
Pulmonary embolism	8	0.22	7	0.76	0.01
Sleep apnea	34	0.92	15	1.63	0.06
**Non-respiratory**					
Hypertension	2,150	58.5	563	61.1	0.14
Ischemic heart disease	1,099	29.9	358	38.9	<0.001
Heart failure	311	8.46	210	22.8	<0.001
Atrial fibrillation	155	4.22	62	6.73	0.001
Diabetes	972	26.4	260	28.2	0.27
Hyperlipidemia	1,420	38.6	348	37.8	0.64
GERD	482	13.1	223	24.2	<0.001
CLD	736	20.0	213	23.1	0.04
CKD	255	6.94	67	7.27	0.72

CLD, chronic liver disease; CKD, chronic kidney disease; COPD, chronic obstructive pulmonary disease; GERD, gastroesophageal reflux disease; and IPF, idiopathic pulmonary fibrosis.

^†^Chi-squared test and Student’s *t*-test.

Simple logistic regression analysis revealed significant comorbidities associated with IPF development, including pulmonary hypertension (crude OR = 21.9, 95% CI = 9.74–49.3), COPD (crude OR = 7.33, 95% CI = 6.26–8.59), asthma (crude OR = 3.75, 95% CI = 3.17–4.44), pulmonary embolism (crude OR = 3.51, 95% CI = 1.27–9.71), heart failure (crude OR = 3.20, 95% CI = 2.64–3.88), GERD (crude OR = 2.12, 95% CI = 1.77–2.53), atrial fibrillation (crude OR = 1.64, 95% CI = 1.21–2.22), ischemic heart disease (crude OR = 1.49, 95% CI = 1.28–1.73), and chronic liver disease (crude OR = 1.20, 95% CI = 1.01–1.43) (Table 2). Moreover, multivariate logistic regression analysis adjusted for sex, age, and comorbidities determined significant comorbidities associated with the IPF development, including pulmonary hypertension (adjusted OR = 16.7, 95% CI = 6.67–41.8), COPD (adjusted OR = 7.43, 95% CI = 6.12–9.02), heart failure (adjusted OR = 1.89, 95% CI = 1.48–2.61), asthma (adjusted OR = 1.69, 95% CI = 1.39–2.07), and GERD (adjusted OR = 1.63, 95% CI = 1.32–2.00).

**Table 2:** Logistic regression analysis for the development of IPF

	IPF			
	Crude OR	95% CI	Adjusted OR^†^	95% CI
**Comorbidity**				
**Respiratory**				
Asthma	3.75	(3.17–4.44)***	1.69	(1.39–2.07)***
COPD	7.33	(6.26–8.59)***	7.43	(6.12–9.02)***
Pulmonary hypertension	21.9	(9.74–49.3)***	16.7	(6.67–41.8)***
Pulmonary embolism	3.51	(1.27–9.71)***	1.60	(0.45–5.76)
Sleep apnea	1.77	(0.96–3.27)	1.32	(0.64–2.75)
**Non-respiratory**				
Hypertension	1.12	(0.96–1.30)	1.06	(0.86–1.34)
Ischemic heart disease	1.49	(1.28–1.73)***	0.96	(0.79–1.17)
Heart failure	3.20	(2.64–3.88)***	1.89	(1.48–2.41)***
Atrial fibrillation	1.64	(1.21–2.22)**	0.92	(0.63–1.34)
Diabetes	1.10	(0.93–1.29)	1.03	(0.84–1.27)
Hyperlipidemia	0.97	(0.83–1.12)	0.87	(0.72–1.05)
GERD	2.12	(1.77–2.53)***	1.63	(1.32–2.00)***
CLD	1.20	(1.01–1.43)*	0.91	(0.74–1.11)
CKD	1.05	(0.80–1.39)	0.76	(0.54–1.05)

CI, confidence interval; CLC, chronic liver disease; CKD, chronic kidney disease; COPD, chronic obstructive pulmonary disease; GERD, gastroesophageal reflux disease; IPF, idiopathic pulmonary fibrosis; and OR, odds ratio.

^†^Multivariable analysis including age, sex, and comorbidity.

*p < 0.05, **p < 0.01, ***p < 0.001.

**Figure 1:**
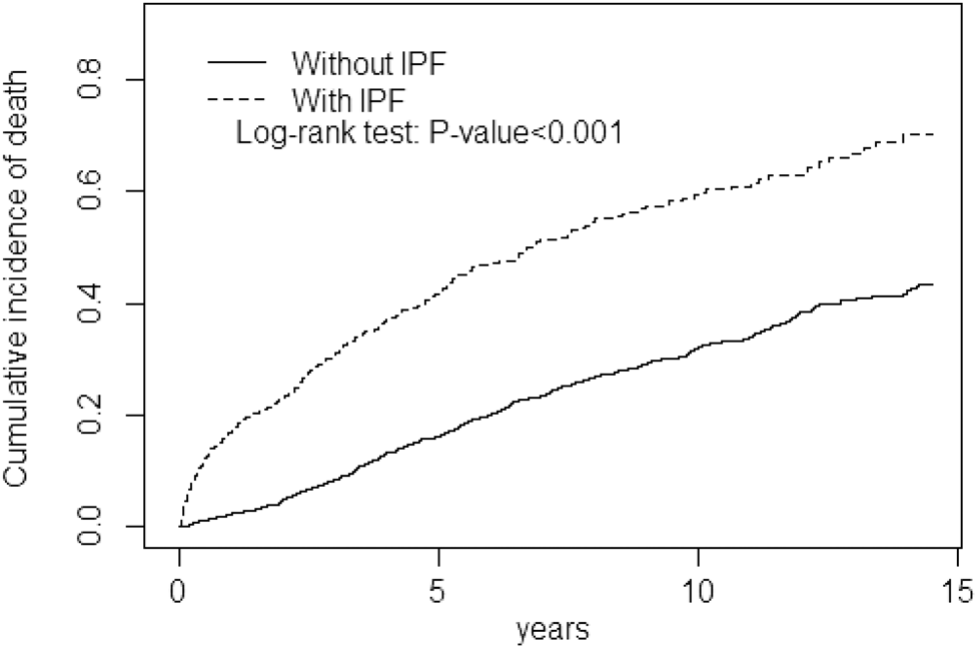
Cumulative incidence of death in IPF cohort and non-IPF cohort.

Furthermore, we calculated the numbers and proportions of pneumonia, acute respiratory failure, chronic respiratory failure, and death from the index date to the end of follow-up between the IPF and non-IPF cohorts (Table 3). The IPF cohort was significantly to have more events of pneumonia (47.6 vs 12.0%, *p* < 0.001), acute respiratory failure (17.8 vs 4.30%, *p* < 0.001), chronic respiratory failure (4.23 vs 0.63%, *p* < 0.001), and death (36.3 vs 15.0%, *p* < 0.001) than the comparative cohort. The time durations of pneumonia, acute respiratory failure, chronic respiratory failure, and death from the index date to the first episode were 2.09 ± 2.98, 3.12 ± 3.62, 3.20 ± 4.03, and 3.27 ± 3.03 years in the IPF cohort and 4.03 ± 3.90, 4.29 ± 4.09, 4.31 ± 4.11, and 4.33 ± 4.11 years in the comparative cohort, respectively. The IPF cohort demonstrated significantly shorter time durations from baseline to the first episode of pneumonia (*p* < 0.001), acute respiratory failure (*p* = 0.007), and death (*p* < 0.001) than the comparative cohort. The cumulative incidence of mortality was significantly higher in the IPF cohort than in the comparative cohort during the follow-up period (log-rank test: *p* < 0.001; Figure 1).

**Table 3:** Proportions and time durations to the first episode of pneumonia, acute respiratory failure, chronic respiratory failure, and death in individuals with and without IPF

	IPF					
	No		Yes			
	*N* = 3,677		*N* = 921			
	*n* (%)	Duration (years)	*n* (%)	Duration (years)	*p*-value^†^	*p*-value^‡^
Pneumonia	440 (12.0)	4.03 ± 3.90	438 (47.6)	2.09 ± 2.98	<0.001	<0.001
Acute respiratory failure	158 (4.30)	4.29 ± 4.09	164 (17.8)	3.12 ± 3.62	<0.001	0.007
Chronic respiratory failure	23 (0.63)	4.31 ± 4.11	39 (4.23)	3.20 ± 4.03	<0.001	0.303
Death	552 (15.0)	4.33 ± 4.11	334 (36.3)	3.27 ± 3.03	<0.001	<0.001

^†^Chi-squared test to compare the proportions of pneumonia, acute respiratory failure, chronic respiratory failure, and death between IPF cohort and the control cohort.

^‡^Student’s *t*-test to compare time durations to the first episode of pneumonia, acute respiratory failure, chronic respiratory failure, and death between IPF cohort and the control cohort.

## 4 Discussion

In the present study, we established a large-scale IPF cohort (*n* = 921) and completed the follow-up. We also set up a well-matched cohort (*n* = 3,677) for comparison. We reported the IPF demographic data (mean age = 69.7 years, male = 58.4%) and identified several important baseline comorbidities and showed their proportions. In addition, we identified the higher risk of major adverse respiratory events and death in the IPF cohort compared with the comparative cohort. Further, we found the time durations to the first episode of pneumonia, acute respiratory failure, and death were significantly shorter in the IPF cohort than in the comparative cohort. These results will serve as a good epidemiological reference.

Epidemiological data of IPF were limited, particularly the results from real-world practice. Recently, Jovanovic et al. collected 3,580 patients with IPF from 11 Central and Eastern European countries (the EMPIRE register study). They reported the top ten comorbidities of IPF, including hypertension (53.0%), diabetes mellitus (24.0%), hyperlipidemia (23.5%), coronary heart disease (23.3%), GERD (21.1%), COPD (16.8%), osteoporosis (13.5%), prostatic hyperplasia (12.1%), pulmonary hypertension (11.7%), and arrhythmia (10.6%). In addition, they reported that 5-year survival was 53.7% in patients without comorbidity, whereas it was 48.4, 47.0, 43.8, and 41.1% in patients with 1, 2, 3, and ≥4 comorbidities, respectively [3]. In another study, Gonzalez-Garcia et al. collected 276 patients with IPF from four Latin American countries. They reported several significant comorbidities, such as pulmonary hypertension (39.9%), systemic hypertension (38.0), GERD (33.9%), diabetes mellitus (20.0%), hyperlipidemia (19.3%), COPD (16.4%), coronary artery disease (15.2%), and hypothyroidism (10.9%) [4]. Although different races and investigative methods may lead to different results, however, the most common comorbidities of IPF could be identified consistently.

IPF carries a poor prognosis, with a median survival of 3.8 years among those aged >65 years in the United States [8]. Many patients die of progressive chronic hypoxemic respiratory failure. Approximately 10–20% of patients with IPF experience an acute exacerbation annually, which may be triggered by a clinical event, usually pneumonia or aspiration, and sometimes idiopathic [9]. Most patients with acute exacerbation die of acute respiratory failure. The present study reported the events, proportions, and time durations of pneumonia, acute respiratory failure, chronic respiratory failure, and death. These events deserve our attention, and we must work to prevent them.

The strength of this study was the establishment of a large-scale IPF cohort with completed follow-up. A well-matched cohort for comparison was another highlight. Moreover, this study was able to reflect a “real world” scenario, in which IPF, all comorbidities, and the major adverse respiratory events were directly diagnosed during medical consultation. Some limitations of our findings should be considered [10,11]. First, ICD codes were used to define IPF and the comorbidities. All diagnoses were dependent on the competence of clinical physicians. The exclusion criteria for the IPF diagnosis were described in previous studies [6,7]. Second, the database did not contain detailed information regarding smoking habits, occupation exposure, and family history, which may be associated with the development of IPF. Third, relevant clinical variables, such as laboratory data, pulmonary function tests, imaging results, and pathological findings, were unavailable. Moreover, the recent development of anti-fibrosing medications may improve IPF outcomes more than that observed in the present study.

## 5 Conclusion

Patients with IPF had several important comorbidities, particularly pulmonary and cardiovascular comorbidities. The duration from diagnosis to the major adverse respiratory events or death was short. Therefore, it is necessary to provide more medical resources to slow down the progression of IPF. In addition, this study may lack more detailed and patient-specific data to comprehensively understand the disease and its associations. We hope that future research can address this limitation.
